# DPL: a comprehensive database on sequences, structures, sources and functions of peptide ligands

**DOI:** 10.1093/database/baaa089

**Published:** 2020-11-20

**Authors:** Fangyu Wang, Ning Li, Chunfeng Wang, Guangxu Xing, Shuai Cao, Qian Xu, Yunshang Zhang, Man Hu, Gaiping Zhang

**Affiliations:** Henan Key Laboratory of Animal Immunology, Henan Academy of Agricultural Sciences, 116# Huayuan Road, Zhengzhou, Henan Province, 450002, China; College of Food Science and Technology, Henan Agricultural University, 63#Agricultural Road, Zhengzhou, Henan Province, 450000, PR China; College of Food Science and Technology, Henan Agricultural University, 63#Agricultural Road, Zhengzhou, Henan Province, 450000, PR China; Department of Gastroenterology, The First Affiliated Hospital of Zhengzhou University, 1# Mianfang Street, Zhengzhou, Henan Province, 450052, China; Henan Key Laboratory of Animal Immunology, Henan Academy of Agricultural Sciences, 116# Huayuan Road, Zhengzhou, Henan Province, 450002, China; College of Food Science and Technology, Henan Agricultural University, 63#Agricultural Road, Zhengzhou, Henan Province, 450000, PR China; Henan Key Laboratory of Animal Immunology, Henan Academy of Agricultural Sciences, 116# Huayuan Road, Zhengzhou, Henan Province, 450002, China; Henan Key Laboratory of Animal Immunology, Henan Academy of Agricultural Sciences, 116# Huayuan Road, Zhengzhou, Henan Province, 450002, China; Henan Key Laboratory of Animal Immunology, Henan Academy of Agricultural Sciences, 116# Huayuan Road, Zhengzhou, Henan Province, 450002, China; Henan Key Laboratory of Animal Immunology, Henan Academy of Agricultural Sciences, 116# Huayuan Road, Zhengzhou, Henan Province, 450002, China; College of Food Science and Technology, Henan Agricultural University, 63#Agricultural Road, Zhengzhou, Henan Province, 450000, PR China

## Abstract

DPL (http://www.peptide-ligand.cn/) is a comprehensive database of peptide ligand (DPL). DPL1.0 holds 1044 peptide ligand entries and provides references for the study of the polypeptide platform. The data were collected from PubMed-NCBI, PDB, APD3, CAMPR3, etc. The lengths of the base sequences are varied from 3 to78. DPL database has 923 linear peptides and 88 cyclic peptides. The functions of peptides collected by DPL are very wide. It includes 540 entries of antiviral peptides (including SARS-CoV-2), 55 entries of signal peptides, 48 entries of protease inhibitors, 45 entries of anti-hypertension, 37 entries of anticancer peptides, etc. There are 270 different kinds of peptide targets. All peptides in DPL have clear binding targets. Most of the peptides and receptors have 3D structures experimentally verified or predicted by CYCLOPS, I-TASSER and SWISS-MODEL. With the rapid development of the COVID-2019 epidemic, this database also collects the research progress of peptides against coronavirus. In conclusion, DPL is a unique resource, which allows users easily to explore the targets, different structures as well as properties of peptides.

Small peptide ligands have been highlighted over the past few decades on account of their particular advantages, such as less cost, little immunogenic responses and more stable physicochemical properties (1, 2). Especially, peptide ligands’ chemical structures are highly compatible with those of the target proteins (3).

Peptide–protein interactions are ubiquitous in living cells and are an important part of the entire protein–protein interaction network. These interactions have attracted increasing attention due to their role in signaling and regulation and are therefore attractive targets for computational structure modeling. Peptide-mediated interactions are a major target for drug design because they are primarily present in signaling and regulatory networks. A reliable data set of non-redundant protein–peptide complexes is an indispensable basis for modeling and design, but current data sets of protein–peptide interactions tend to be biased towards specific types of interactions or limited to interactions with small ligands (4).

Peptide–protein interactions can happen in a lot of interaction networks and only need a small interface (5). As a result, these small molecules and inhibitory peptides are attractive drug targets (6, 7). This means that the synthetic peptides can be designed to change the specific interaction of disease or other signal pathways (8). Besides the peptide structure stored in the Protein Data Bank (PDB) (9), there are about 20 new items to show the interaction of small peptides each month (10). As the new and interesting structure of the protein–peptide complex is growing, our understanding of the interaction mechanism between protein and peptide also should be improved. Peptides tend to bind at the largest pocket available on the protein surface (11). To understand and analyze the interaction mechanism of protein and peptide, establishing a reliable database of peptide ligands is necessary. There are many protein–peptide interaction database based on sequences, such as Phospho.ELM (12), DOMINO (13), Pep Bank (14), SCANSITE (15), APD (16), BIOPEP (17) and ASPD (18). However, the database of peptide ligand (DPL) is a set of 1044 peptides for non-redundant protein–peptide complexes based on different binding targets.

Previous studies have reported combined with multiple peptide or protein with the heterogeneity of the structures of the domain (e.g. there are at least 13 different types of peptides was reported to SH3 domain structure (19)). For a detailed analysis of similar proteins and the interaction between different peptides, it needs a lot of data on the structure and ligand of protein–peptide complexes. To solve this problem, we created a DPL.

This DPL project has built a clear target of the peptide ligands database through the literature summary, including specific peptide information 1044, which provides a reference for the study of the polypeptide platform. All the peptides have a clear binding target, have to be experimentally verified and collect the 3D structures of all ligands and receptors. DPL is a unique resource, which allows users easily to explore the different structures as well as properties of peptides.

## Construction and content

### Data collection

Database of peptide ligand in DPL was collected from PubMed-NCBI, PDB, the 3rd version of the APD (APD3) and Collection of Anti-Microbial Peptides(CAMPR3) by using keywords such as ‘antibacterial peptide’, ‘antiviral peptide’, ‘antifungal peptide’, ‘antiparasitic peptide’, ‘anticancer peptide’, ‘insecticidal peptide’, ‘anti-inflammatory peptides’, ‘anti-toxic peptides’, ‘protease inhibitors peptides’, ‘antioxidant peptides’ ‘anti-hypertension peptides’ or ‘signal peptides’ (12–18).

### Utility

The main web page of DPL contains the following aspects: Home, Database search, Tools, News, Links, Publications and Our team.

### Home page

The use and main criteria of DPL are introduced on the Home page briefly. DPL is a specialized database for the collection of targeted binding polypeptides. There are three main criteria for data collection in this database: the peptides have a clear binding target; these peptides have to be experimentally verified, and this database strives to collect the 3D structure of all ligands and receptors. The prediction method of the structure of peptide using the web tools as CYCLOPS, I-TASSER or SWISS-MODEL (20–22).

### Search page

A quick search was constructed on the search page through some appropriate keywords, such as peptide name, ID, sequence, function or receptor name, function. The search will ambiguously match any residue in the peptide name, ID, sequence, function or receptor name, function. To get more accurate results, please try to enter more detailed search terms.

## Results and discussion

### Sequences

**Figure 1.**
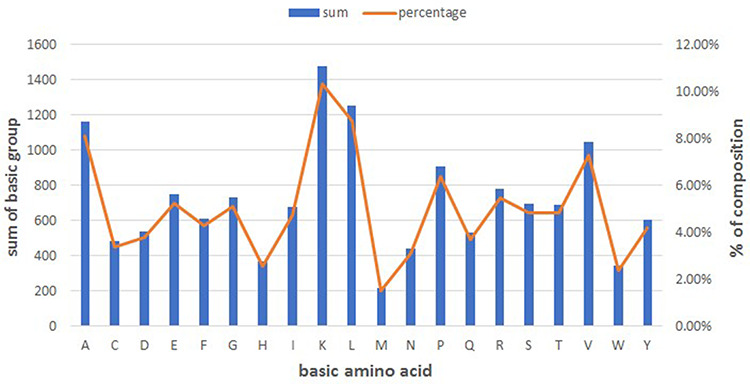
Basic amino acid distribution in DPL.

Figure 1 summarizes the basic amino acid distribution. As shown, alanine, lysine, leucine and valine make up the predominant composition in peptides (See Figure 1).

**Figure 2.**
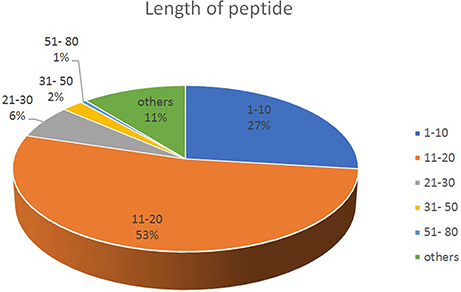
The proportion of different length of peptide in DPL.

The length of the base sequence varied from 3 to78. As the length of the peptide chain is different, the proportion is different. The most proportion of peptide is in the length of 11–20 (53%), followed by 1–10 (27%); 51–80 proportion is at least (1%) (See Figure 2).

### The type of peptide

**Figure 3.**
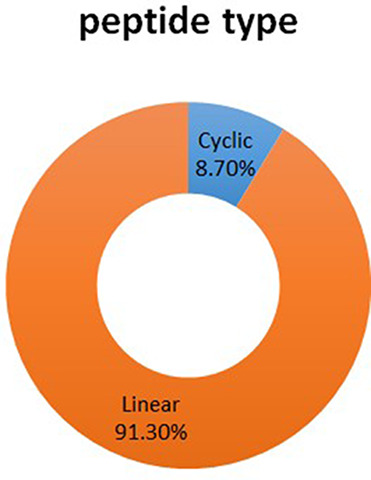
The proportion of two kinds of peptide structure in DPL.

The DPL database has 1044 entries in total. There are two kinds of peptide structures, such as linear peptide and cyclic peptide, respectively, in which linear peptide has 923 entries, accounts for 91.30%; cyclic peptide has 88 entries, accounted for 8.70% (See Figure 3).

### Function

The function of peptide collected by DPL database is very wide, such as ‘antibacterial peptide’, ‘antiviral peptide’, ‘antifungal peptide’, ‘antiparasitic peptide’, ‘anticancer peptide‘, ‘insecticidal peptide‘, ‘anti-inflammatory peptides’, ‘anti-toxic peptides’, ‘protease inhibitors peptides’, ‘antioxidant peptides’, ‘anti-hypertension peptides’, ‘signal peptides’, etc.

**Figure 4.**
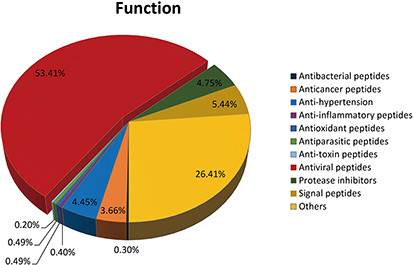
The functions of peptides in DPL.

A total of 540 kinds of antiviral peptides account for 53.14%; 267 kinds of others accounted for 26.41%; 55 kinds of signal peptides accounted for 5.44%; 48 kinds of protease inhibitors accounted for 4.75%; 45 kinds of anti-hypertension account for 4.45%; 37 kinds of anticancer peptides accounted for 3.66%; 5 kinds of antioxidant peptides accounted for 0.49%; 5 kinds of anti-parasitic peptides accounted for 0.49%; 3 kinds of antibacterial peptides accounted for 0.30%; 4 kinds of anti-inflammatory peptides accounted for 0.40%; 2 kinds of anti-toxin peptides accounting for 0.20% (See Figure 4).

### The source of peptide structures

**Figure 5.**
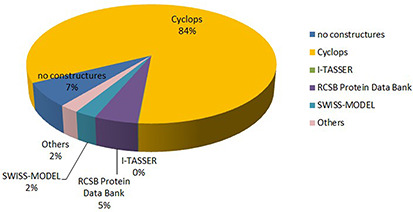
The sources of peptide structure in DPL.

The structure of peptide has different sources, such as CYCLOPS (846 entries), I-TASSER (1 entry), SWISS-MODEL (25 entries), RCSB Protein Data Bank (50 entries), no structure (67 entries), etc. The proportion of different sources of the structure is shown in Figure 5.

### Targets of peptide

There are 270 different kinds of peptide targets.

### Peptide ligands for COVID-2019

With the rapid development of the COVID-2019 epidemic, this database also collects and organizes the research progress of peptides against coronavirus (Table 1). Detailed information such as peptide sequences, targets and research literature are recorded in this database.

**Table 1.** Detailed information of some peptides against coronavirus collected in DPL

DPL_ID	Sequence	Source	Virus	IC50	Model
1028	GYHLMSFPQAAPHGVVFLHVTW	S2	SARS	∼2 μM	VeroE6, L2
1030	GYFVQDDGEWKFTGSSYYY	S2	MHV	4 μM	VeroE6, L2
1031	LTQINTTLLQDLTYEMLSLQQVVKALNESYIDLKEL	HR2	MERS	∼3.013 μM	293T
1032	SIPNFGSLTQINTTLLDLTYEMLSLQQVVKALNESYIDLKELGNY	HR2	MERS	∼0.5 μM	293T/EGFP + HUH-7
1033	SLTQINTTLLDLTYEMLSLQQVVKALNESYIDLKELY	HR2	MERS	∼0.97 ± 0.15 μM	293T/EGFP + HUH-7
				∼0.6 μM	Vero
				∼0.6 μM	Calu-3
				13.9 μM	HFL
1034	SLTQINTTLLDLEYEMRSLQQVVKALNESYIDLKEL	HR2	MERS	∼0.85 ± 0.08 μM	293T/EGFP + HUH-7
1035	SLTQINTTLLDLEYEMKKLEEVVKKLEESYIDLKEL	HR2	MERS	∼0.55 ± 0.04 μM	293T + HUH-7
1036	NGAICWGPCPTAFRQIGNCGHRKVRCCKIR	β-4	MERS	5 μM	Mice
1037	FGGASCCLYCRCHIDHPNPKGFCDLKGKY	Nsp10	SARS	160 μM	*E. coli*
1038	GGASCCLYCRCH	Nsp10	SARS	160 μM	*E. coli*
1039	LFRLIKSLIKRLVSAFK	AMP	SARS	7.15 μg/ml	MDCK
1040	HVTTTFAPPPPR	pAMN	TGEV	11 μg/ml	ST
1041	SVVPSKATWGFA	pAMN	TGEV	15 μg/ml	ST
1042	YKYRYL	RBD	SARS	KD = 46 μM	VeroE6
1043	PSSKRFQPFQQFGRDVSDFT	S	SARS		293T
1044	CANLLLQYGSFCTQLNRALSGIA	S	SARS		293T

The most influential databases in this field are PDB, APD3, CAMP3, etc. This resource is powered by the PDB archive-information about the 3D shapes of proteins, nucleic acids and complex assemblies that help students and researchers understand all aspects of biomedicine and agriculture, from protein synthesis to health and disease (23). APD3 reported 2619 peptides. New web pages for FAQs, interesting AMP discovery timeline, classification, nomenclature, AMP facts, My tools, Sequence download and APD News have been created (16). A unified peptide classification system has been proposed and introduced in APD. Besides, the prediction interface has been improved and additional peptide properties can be calculated in APD. CAMPR3 has been created to expand and accelerate antimicrobial peptide family based studies. Antimicrobial peptides have family specific sequence composition which can be mined to discover and design novel AMPs (24). In a word, each database has its advantages and disadvantages.

Peptide ligands can simulate protein–protein interactions and have large binding interfaces with receptors; thus, they possess much higher binding affinity and specificity than small-molecule ligands. Peptides offer a potent resource for targeted drug delivery. Compared to protein ligands, peptides have many advantages, including better penetration, ease of synthesis and lower immunogenicity and cost. Large-scale synthesis of peptides presents a convenient and economical option for drug use; also, due to the abundant chemical groups in peptides, they are suitable for manipulation.

However, this article briefly introduces the DPL database to collect many peptide ligands for users. This DPL database has built a clear target of the peptide ligands database through the literature summary, including specific peptide information 1044, which provides a reference for the study of the polypeptide platform. All the information of peptides and receptors collected in DPL provides material for molecular docking and virtual screening in future. DPL database will build the virtual peptide library through the computer program and set up a molecular docking platform and analyze the differences between global and local molecular docking results in next version. All the peptides and targets have a clear binding target. 16 items anti-coronavirus peptides also were added in DPL, it provides technical support for the target screening and research of vaccines and drugs. DPL database is a unique resource and still being updated, which allows users easily to explore the different structures as well as properties of peptides.

## Conclusion

With 1044 entries, DPL is an open-access, manually curated database with a clear binding target, be experimentally verified, and collect the 3D structure of all peptide ligands and receptors. To the best of authors’ knowledge, DPL is the only database available to the public, which provides comprehensive information on DPL, especially provides structures of all peptides. User-friendly interfaces have been established to facilitate peptides searching, browsing and alignment. DPL should help promote our understanding of peptide ligands and should provide a valuable resource for the development of peptide application. We believe that the DPL will be very useful for scientists in peptide research.

## Availability

DPL can be freely accessed at the URL: http://www.peptide-ligand.cn/. Users are invited to submit their suggestions and peptide sequences by subscription to the site. The database will be expanded and improved by the authors in the course of time.
